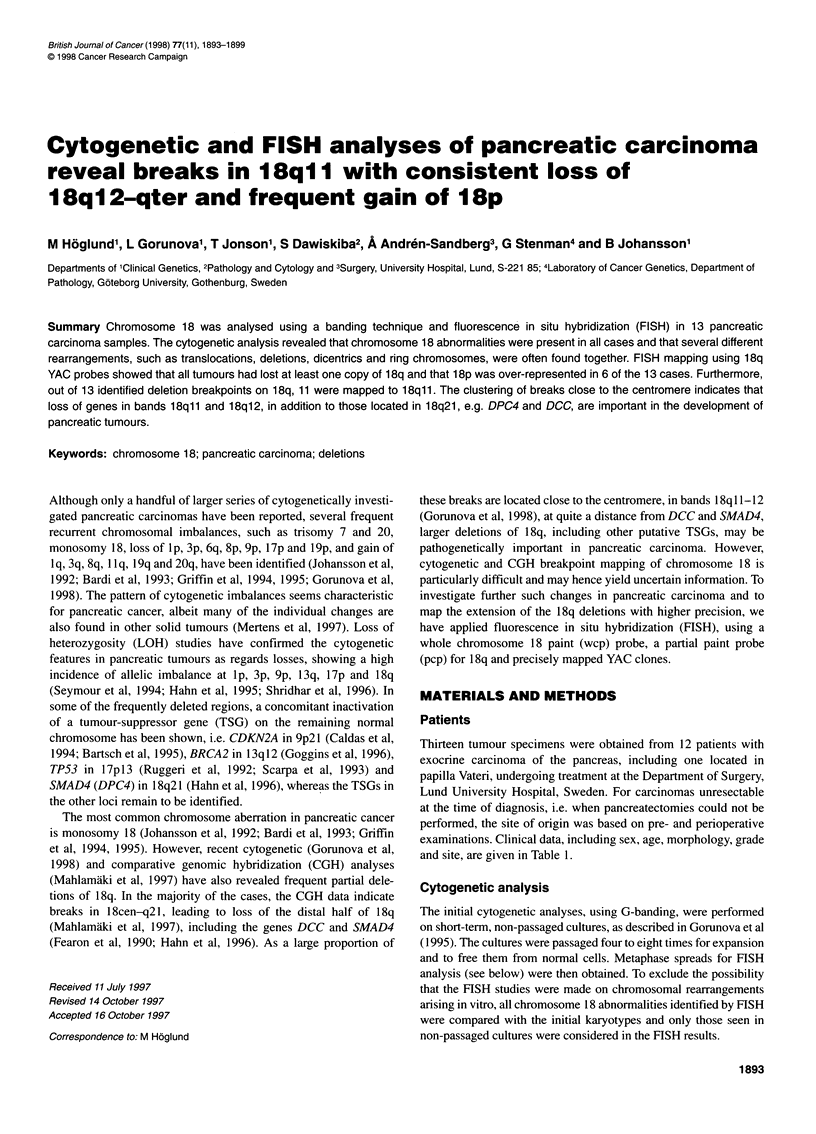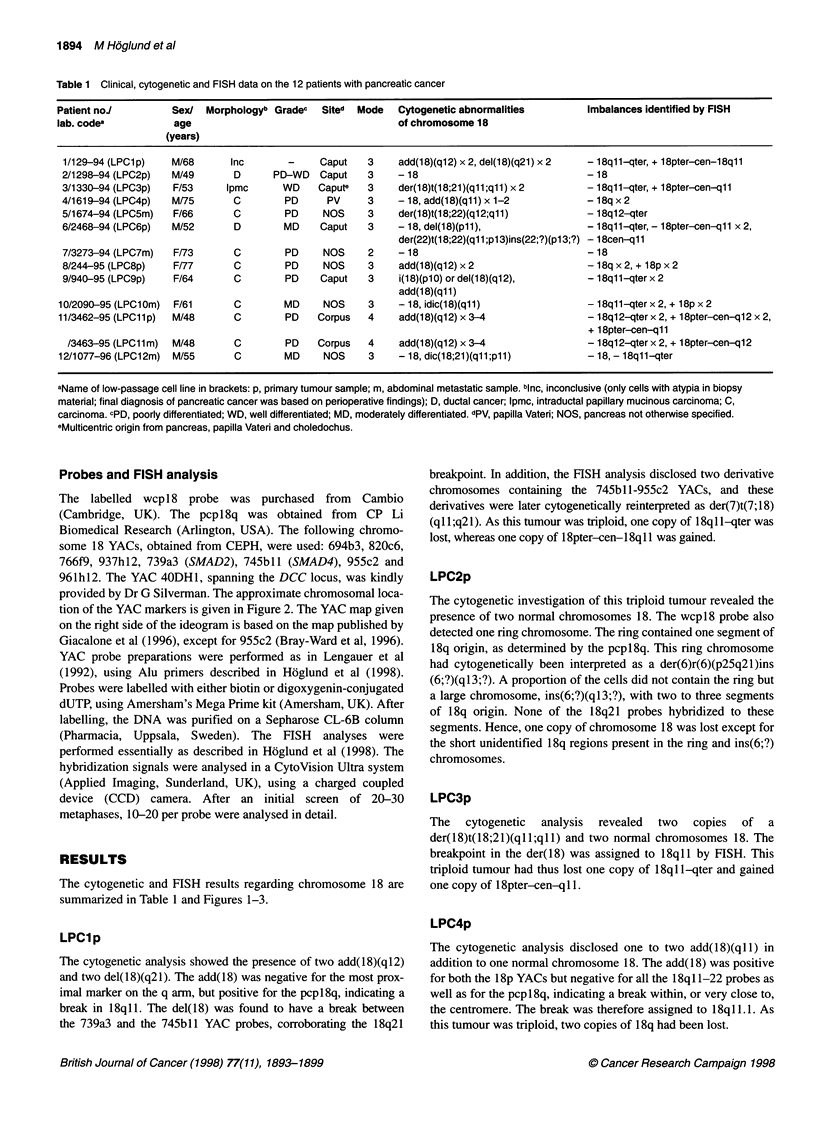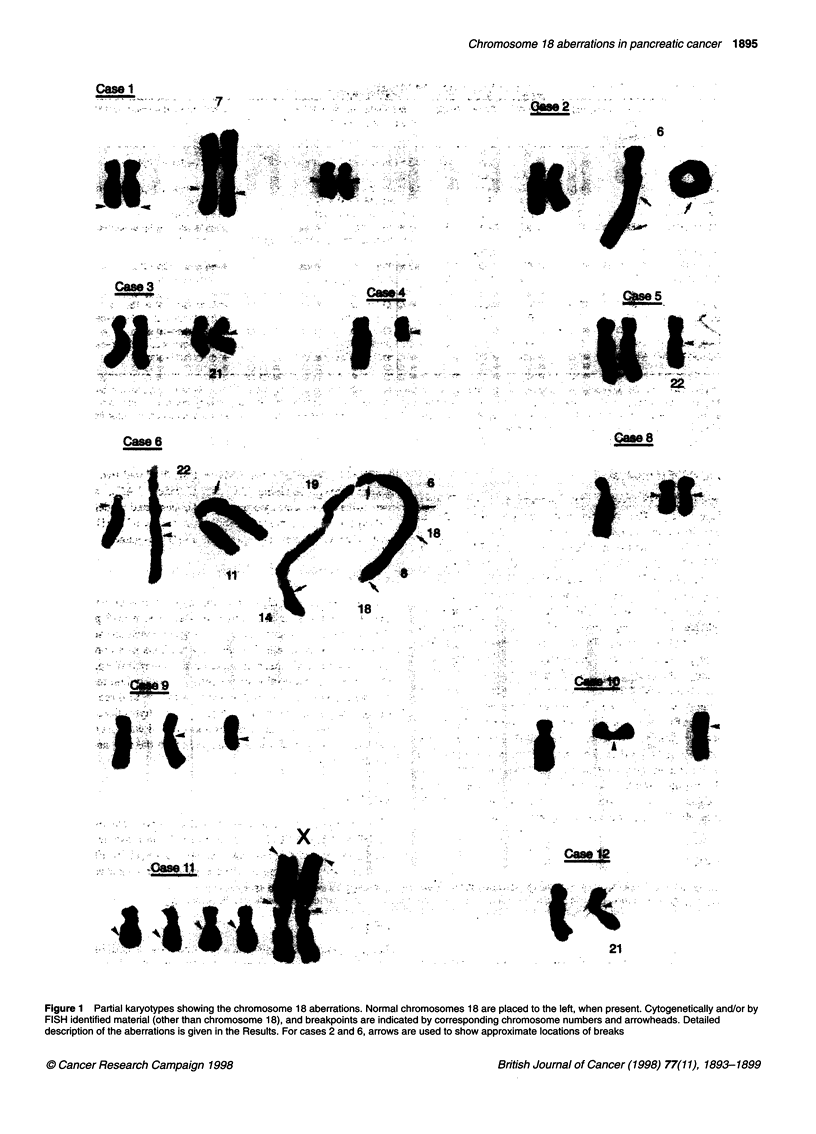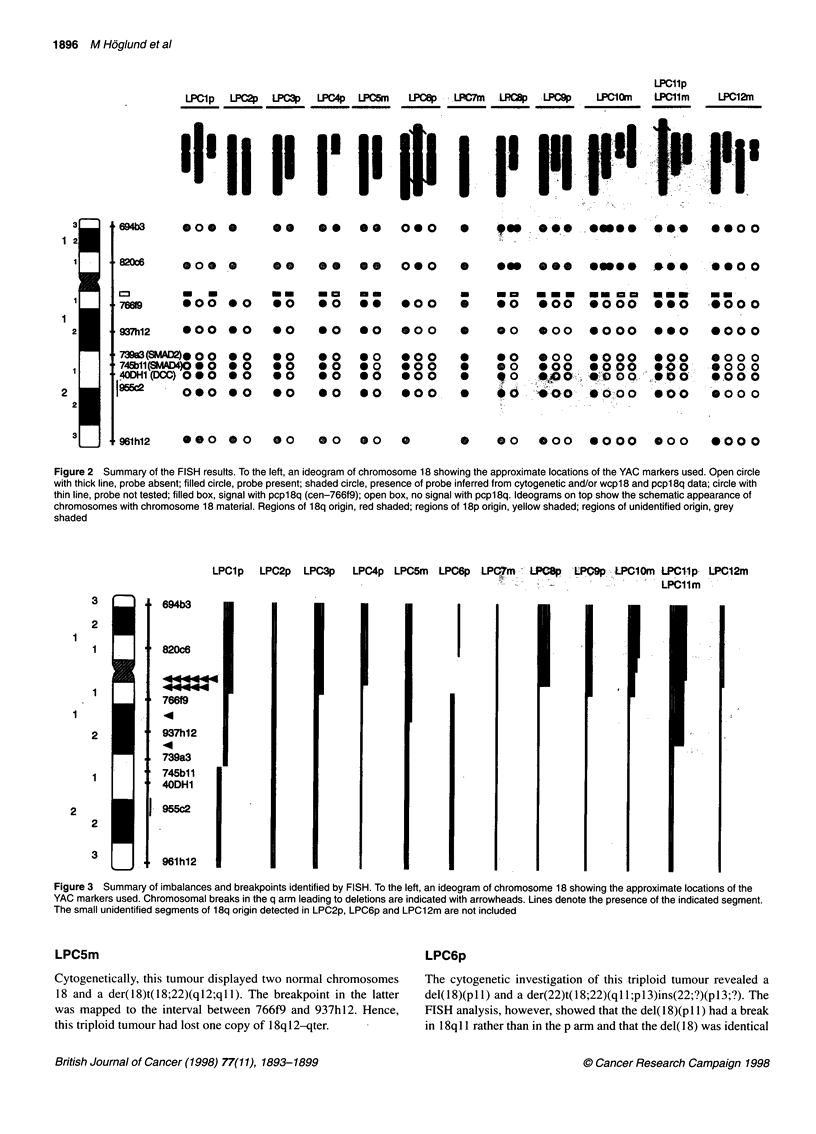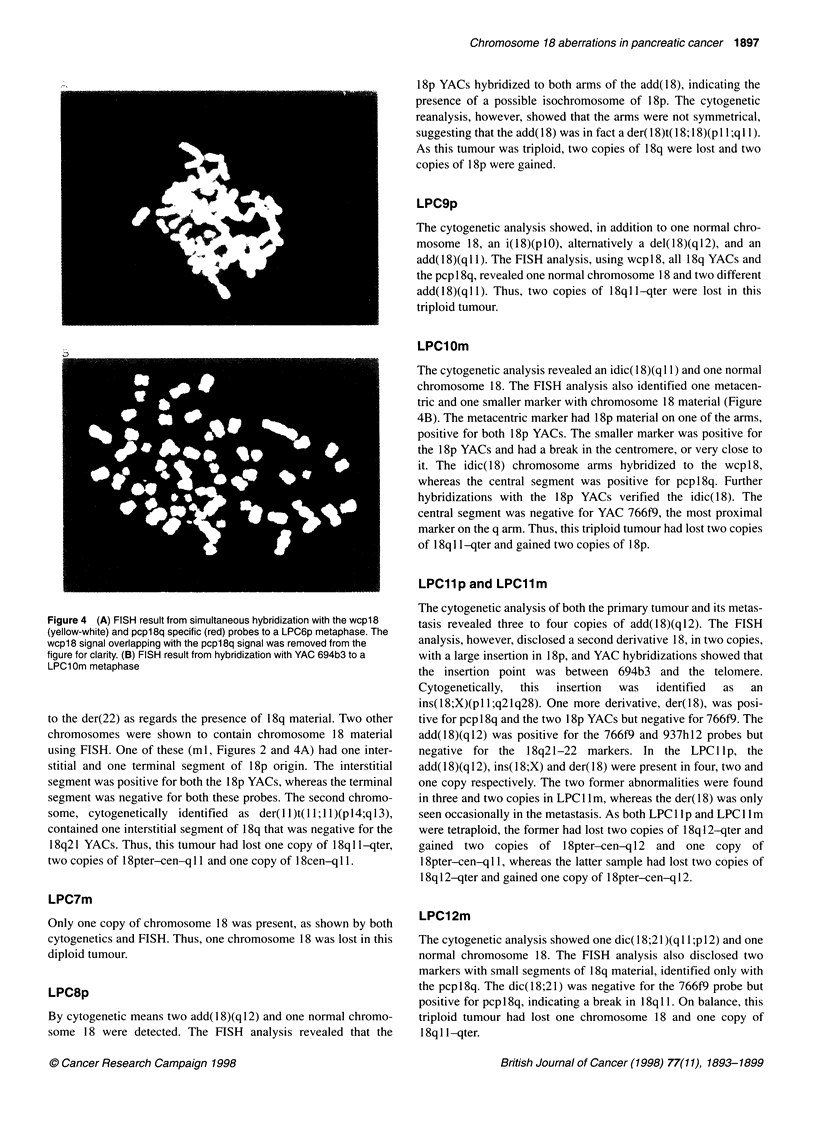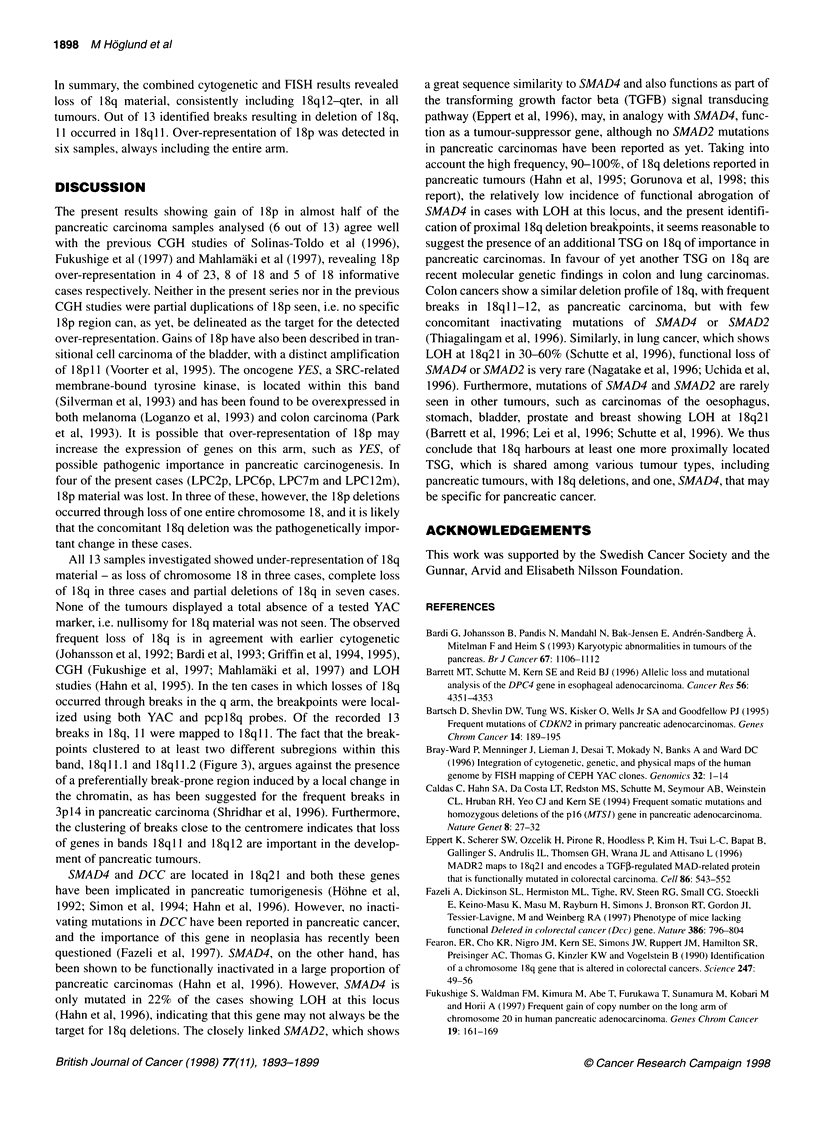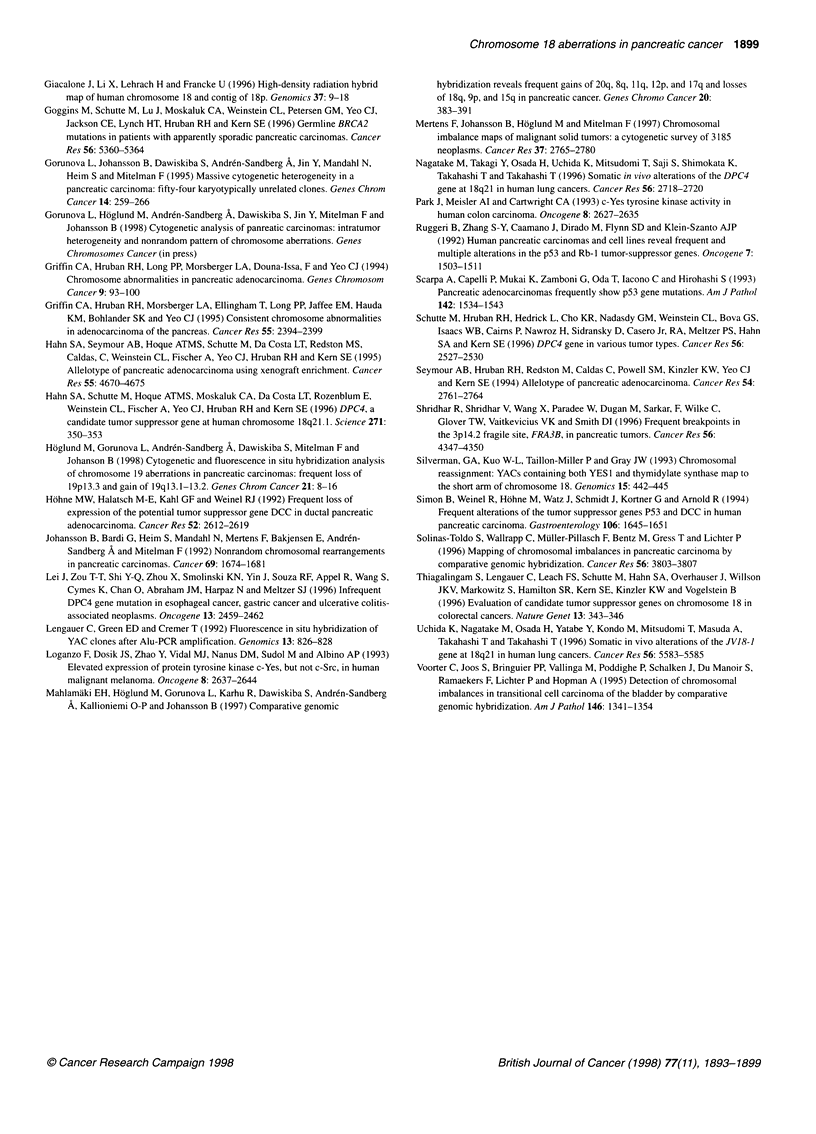# Cytogenetic and FISH analyses of pancreatic carcinoma reveal breaks in 18q11 with consistent loss of 18q12-qter and frequent gain of 18p.

**DOI:** 10.1038/bjc.1998.315

**Published:** 1998-06

**Authors:** M. HÃ¶glund, L. Gorunova, T. Jonson, S. Dawiskiba, A. AndrÃ©n-Sandberg, G. Stenman, B. Johansson

**Affiliations:** Department of Clinical Genetics, University Hospital, Lund, Sweden.

## Abstract

**Images:**


*British Journal of Cancer* (1998) **77**(11), 1893–1899
© 1998 Cancer Research Campaign

### Cytogenetic and FISH analyses of pancreatic carcinoma reveal breaks in 18q11 with consistent loss of 18q12–qter and frequent gain of 18p

**M Höglund[1], L Gorunova[1], T Jonson[1], S Dawiskiba[2], Å Andrén-Sandberg[3], G Stenman[4] and B Johansson[1]**

Departments of [1]Clinical Genetics, [2]Pathology and Cytology and [3]Surgery, University Hospital, Lund, S-221 85; [4]Laboratory of Cancer Genetics, Department of Pathology, Göteborg University, Gothenburg, Sweden

**Summary** Chromosome 18 was analysed using a banding technique and fluorescence in situ hybridization (FISH) in 13 pancreatic carcinoma samples. The cytogenetic analysis revealed that chromosome 18 abnormalities were present in all cases and that several different rearrangements, such as translocations, deletions, dicentrics and ring chromosomes, were often found together. FISH mapping using 18q YAC probes showed that all tumours had lost at least one copy of 18q and that 18p was over-represented in 6 of the 13 cases. Furthermore, out of 13 identified deletion breakpoints on 18q, 11 were mapped to 18q11. The clustering of breaks close to the centromere indicates that loss of genes in bands 18q11 and 18q12, in addition to those located in 18q21, e.g. *DPC4* and *DCC*, are important in the development of pancreatic tumours.

**Keywords:** chromosome 18; pancreatic carcinoma; deletions

Although only a handful of larger series of cytogenetically investigated pancreatic carcinomas have been reported, several frequent recurrent chromosomal imbalances, such as trisomy 7 and 20, monosomy 18, loss of 1p, 3p, 6q, 8p, 9p, 17p and 19p, and gain of 1q, 3q, 8q, 11q, 19q and 20q, have been identified (Johansson et al, 1992; Bardi et al, 1993; Griffin et al, 1994, 1995; Gorunova et al, 1998). The pattern of cytogenetic imbalances seems characteristic for pancreatic cancer, albeit many of the individual changes are also found in other solid tumours (Mertens et al, 1997). Loss of heterozygosity (LOH) studies have confirmed the cytogenetic features in pancreatic tumours as regards losses, showing a high incidence of allelic imbalance at 1p, 3p, 9p, 13q, 17p and 18q (Seymour et al, 1994; Hahn et al, 1995; Shridhar et al, 1996). In some of the frequently deleted regions, a concomitant inactivation of a tumour-suppressor gene (TSG) on the remaining normal chromosome has been shown, i.e. *CDKN2A* in 9p21 (Caldas et al, 1994; Bartsch et al, 1995), *BRCA2* in 13q12 (Goggins et al, 1996), *TP53* in 17p13 (Ruggeri et al, 1992; Scarpa et al, 1993) and *SMAD4* (*DPC4*) in 18q21 (Hahn et al, 1996), whereas the TSGs in the other loci remain to be identified.

The most common chromosome aberration in pancreatic cancer is monosomy 18 (Johansson et al, 1992; Bardi et al, 1993; Griffin et al, 1994, 1995). However, recent cytogenetic (Gorunova et al, 1998) and comparative genomic hybridization (CGH) analyses (Mahlamäki et al, 1997) have also revealed frequent partial deletions of 18q. In the majority of the cases, the CGH data indicate breaks in 18cen–q21, leading to loss of the distal half of 18q (Mahlamäki et al, 1997), including the genes *DCC* and *SMAD4* (Fearon et al, 1990; Hahn et al, 1996). As a large proportion of these breaks are located close to the centromere, in bands 18q11–12 (Gorunova et al, 1998), at quite a distance from *DCC* and *SMAD4*, larger deletions of 18q, including other putative TSGs, may be pathogenetically important in pancreatic carcinoma. However, cytogenetic and CGH breakpoint mapping of chromosome 18 is particularly difficult and may hence yield uncertain information. To investigate further such changes in pancreatic carcinoma and to map the extension of the 18q deletions with higher precision, we have applied fluorescence in situ hybridization (FISH), using a whole chromosome 18 paint (wcp) probe, a partial paint probe (pcp) for 18q and precisely mapped YAC clones.

*Received 11 July 1997*
*Revised 14 October 1997*
*Accepted 16 October 1997*

*Correspondence to:* M Höglund

### MATERIALS AND METHODS

### Patients

Thirteen tumour specimens were obtained from 12 patients with exocrine carcinoma of the pancreas, including one located in papilla Vateri, undergoing treatment at the Department of Surgery, Lund University Hospital, Sweden. For carcinomas unresectable at the time of diagnosis, i.e. when pancreatectomies could not be performed, the site of origin was based on pre- and perioperative examinations. Clinical data, including sex, age, morphology, grade and site, are given in Table 1.

### Cytogenetic analysis

The initial cytogenetic analyses, using G-banding, were performed on short-term, non-passaged cultures, as described in Gorunova et al (1995). The cultures were passaged four to eight times for expansion and to free them from normal cells. Metaphase spreads for FISH analysis (see below) were then obtained. To exclude the possibility that the FISH studies were made on chromosomal rearrangements arising in vitro, all chromosome 18 abnormalities identified by FISH were compared with the initial karyotypes and only those seen in non-passaged cultures were considered in the FISH results.

**Table 1** Clinical, cytogenetic and FISH data on the 12 patients with pancreatic cancer

| Patient no./ lab. code[a] | Sex/ age (years) | Morphology[b] | Grade[c] | Site[d] | Mode | Cytogenetic abnormalities of chromosome 18 | Imbalances identified by FISH |
|---|---|---|---|---|---|---|---|
| 1/129–94 (LPC1p) | M/68 | Inc | – | Caput | 3 | add(18)(q12) × 2, del(18)(q21) × 2 | – 18q11–qter, + 18pter–cen–18q11 |
| 2/1298–94 (LPC2p) | M/49 | D | PD–WD | Caput | 3 | – 18 | – 18 |
| 3/1330–94 (LPC3p) | F/53 | Ipmc | WD | Caput[e] | 3 | der(18)t(18;21)(q11;q11) × 2 | – 18q11–qter, + 18pter–cen–q11 |
| 4/1619–94 (LPC4p) | M/75 | C | PD | PV | 3 | – 18, add(18)(q11) × 1–2 | – 18q × 2 |
| 5/1674–94 (LPC5m) | F/66 | C | PD | NOS | 3 | der(18)t(18;22)(q12;q11) | – 18q12–qter |
| 6/2468–94 (LPC6p) | M/52 | D | MD | Caput | 3 | – 18, del(18)(p11), der(22)t(18;22)(q11;p13)ins(22;?)(p13;?) | – 18q11–qter, – 18pter–cen–q11 × 2, – 18cen–q11 |
| 7/3273–94 (LPC7m) | F/73 | C | PD | NOS | 2 | – 18 | – 18 |
| 8/244–95 (LPC8p) | F/77 | C | PD | NOS | 3 | add(18)(q12) × 2 | – 18q × 2, + 18p × 2 |
| 9/940–95 (LPC9p) | F/64 | C | PD | Caput | 3 | i(18)(p10) or del(18)(q12), add(18)(q11) | – 18q11–qter × 2 |
| 10/2090–95 (LPC10m) | F/61 | C | MD | NOS | 3 | – 18, idic(18)(q11) | – 18q11–qter × 2, + 18p × 2 |
| 11/3462–95 (LPC11p) | M/48 | C | PD | Corpus | 4 | add(18)(q12) × 3–4 | – 18q12–qter × 2, + 18pter–cen–q12 × 2, + 18pter–cen–q11 |
| /3463–95 (LPC11m) | M/48 | C | PD | Corpus | 4 | add(18)(q12) × 3–4 | – 18q12–qter × 2, + 18pter–cen–q12 |
| 12/1077–96 (LPC12m) | M/55 | C | MD | NOS | 3 | – 18, dic(18;21)(q11;p11) | – 18, – 18q11–qter |

[a]Name of low-passage cell line in brackets: p, primary tumour sample; m, abdominal metastatic sample. [b]Inc, inconclusive (only cells with atypia in biopsy material; final diagnosis of pancreatic cancer was based on perioperative findings); D, ductal cancer; Ipmc, intraductal papillary mucinous carcinoma; C, carcinoma. [c]PD, poorly differentiated; WD, well differentiated; MD, moderately differentiated. [d]PV, papilla Vateri; NOS, pancreas not otherwise specified. [e]Multicentric origin from pancreas, papilla Vateri and choledochus.

### Probes and FISH analysis

The labelled wcp18 probe was purchased from Cambio (Cambridge, UK). The pcp18q was obtained from CP Li Biomedical Research (Arlington, USA). The following chromosome 18 YACs, obtained from CEPH, were used: 694b3, 820c6, 766f9, 937h12, 739a3 (*SMAD2*), 745b11 (*SMAD4*), 955c2 and 961h12. The YAC 40DH1, spanning the *DCC* locus, was kindly provided by Dr G Silverman. The approximate chromosomal location of the YAC markers is given in Figure 2. The YAC map given on the right side of the ideogram is based on the map published by Giacalone et al (1996), except for 955c2 (Bray-Ward et al, 1996). YAC probe preparations were performed as in Lengauer et al (1992), using Alu primers described in Höglund et al (1998). Probes were labelled with either biotin or digoxygenin-conjugated dUTP, using Amersham's Mega Prime kit (Amersham, UK). After labelling, the DNA was purified on a Sepharose CL-6B column (Pharmacia, Uppsala, Sweden). The FISH analyses were performed essentially as described in Höglund et al (1998). The hybridization signals were analysed in a CytoVision Ultra system (Applied Imaging, Sunderland, UK), using a charged coupled device (CCD) camera. After an initial screen of 20–30 metaphases, 10–20 per probe were analysed in detail.

### RESULTS

The cytogenetic and FISH results regarding chromosome 18 are summarized in Table 1 and Figures 1–3.

### LPC1p

The cytogenetic analysis showed the presence of two add(18)(q12) and two del(18)(q21). The add(18) was negative for the most proximal marker on the q arm, but positive for the pcp18q, indicating a break in 18q11. The del(18) was found to have a break between the 739a3 and the 745b11 YAC probes, corroborating the 18q21 breakpoint. In addition, the FISH analysis disclosed two derivative chromosomes containing the 745b11-955c2 YACs, and these derivatives were later cytogenetically reinterpreted as der(7)t(7;18)(q11;q21). As this tumour was triploid, one copy of 18q11–qter was lost, whereas one copy of 18pter–cen–18q11 was gained.

### LPC2p

The cytogenetic investigation of this triploid tumour revealed the presence of two normal chromosomes 18. The wcp18 probe also detected one ring chromosome. The ring contained one segment of 18q origin, as determined by the pcp18q. This ring chromosome had cytogenetically been interpreted as a der(6)r(6)(p25q21)ins(6;?)(q13;?). A proportion of the cells did not contain the ring but a large chromosome, ins(6;?)(q13;?), with two to three segments of 18q origin. None of the 18q21 probes hybridized to these segments. Hence, one copy of chromosome 18 was lost except for the short unidentified 18q regions present in the ring and ins(6;?) chromosomes.

### LPC3p

The cytogenetic analysis revealed two copies of a der(18)t(18;21)(q11;q11) and two normal chromosomes 18. The breakpoint in the der(18) was assigned to 18q11 by FISH. This triploid tumour had thus lost one copy of 18q11–qter and gained one copy of 18pter–cen–q11.

### LPC4p

The cytogenetic analysis disclosed one to two add(18)(q11) in addition to one normal chromosome 18. The add(18) was positive for both the 18p YACs but negative for all the 18q11–22 probes as well as for the pcp18q, indicating a break within, or very close to, the centromere. The break was therefore assigned to 18q11.1. As this tumour was triploid, two copies of 18q had been lost.

© Cancer Research Campaign 1998

**Figure 1** Partial karyotypes showing the chromosome 18 aberrations. Normal chromosomes 18 are placed to the left, when present. Cytogenetically and/or by FISH identified material (other than chromosome 18), and breakpoints are indicated by corresponding chromosome numbers and arrowheads. Detailed description of the aberrations is given in the Results. For cases 2 and 6, arrows are used to show approximate locations of breaks

© Cancer Research Campaign 1998

**Figure 2** Summary of the FISH results. To the left, an ideogram of chromosome 18 showing the approximate locations of the YAC markers used. Open circle with thick line, probe absent; filled circle, probe present; shaded circle, presence of probe inferred from cytogenetic and/or wcp18 and pcp18q data; circle with thin line, probe not tested; filled box, signal with pcp18q (cen–766f9); open box, no signal with pcp18q. Ideograms on top show the schematic appearance of chromosomes with chromosome 18 material. Regions of 18q origin, red shaded; regions of 18p origin, yellow shaded; regions of unidentified origin, grey shaded

**Figure 3** Summary of imbalances and breakpoints identified by FISH. To the left, an ideogram of chromosome 18 showing the approximate locations of the YAC markers used. Chromosomal breaks in the q arm leading to deletions are indicated with arrowheads. Lines denote the presence of the indicated segment. The small unidentified segments of 18q origin detected in LPC2p, LPC6p and LPC12m are not included

### LPC5m

Cytogenetically, this tumour displayed two normal chromosomes 18 and a der(18)t(18;22)(q12;q11). The breakpoint in the latter was mapped to the interval between 766f9 and 937h12. Hence, this triploid tumour had lost one copy of 18q12–qter.

### LPC6p

The cytogenetic investigation of this triploid tumour revealed a del(18)(p11) and a der(22)t(18;22)(q11;p13)ins(22;?)(p13;?). The FISH analysis, however, showed that the del(18)(p11) had a break in 18q11 rather than in the p arm and that the del(18) was identical

© Cancer Research Campaign 1998

**Figure 4** (**A**) FISH result from simultaneous hybridization with the wcp18 (yellow-white) and pcp18q specific (red) probes to a LPC6p metaphase. The wcp18 signal overlapping with the pcp18q signal was removed from the figure for clarity. (**B**) FISH result from hybridization with YAC 694b3 to a LPC10m metaphase to the der(22) as regards the presence of 18q material. Two other chromosomes were shown to contain chromosome 18 material using FISH. One of these (m1, Figures 2 and 4A) had one interstitial and one terminal segment of 18p origin. The interstitial segment was positive for both the 18p YACs, whereas the terminal segment was negative for both these probes. The second chromosome, cytogenetically identified as der(11)t(11;11)(p14;q13), contained one interstitial segment of 18q that was negative for the 18q21 YACs. Thus, this tumour had lost one copy of 18q11–qter, two copies of 18pter–cen–q11 and one copy of 18cen–q11.

### LPC7m

Only one copy of chromosome 18 was present, as shown by both cytogenetics and FISH. Thus, one chromosome 18 was lost in this diploid tumour.

### LPC8p

By cytogenetic means two add(18)(q12) and one normal chromosome 18 were detected. The FISH analysis revealed that the 18p YACs hybridized to both arms of the add(18), indicating the presence of a possible isochromosome of 18p. The cytogenetic reanalysis, however, showed that the arms were not symmetrical, suggesting that the add(18) was in fact a der(18)t(18;18)(p11;q11). As this tumour was triploid, two copies of 18q were lost and two copies of 18p were gained.

### LPC9p

The cytogenetic analysis showed, in addition to one normal chromosome 18, an i(18)(p10), alternatively a del(18)(q12), and an add(18)(q11). The FISH analysis, using wcp18, all 18q YACs and the pcp18q, revealed one normal chromosome 18 and two different add(18)(q11). Thus, two copies of 18q11–qter were lost in this triploid tumour.

### LPC10m

The cytogenetic analysis revealed an idic(18)(q11) and one normal chromosome 18. The FISH analysis also identified one metacentric and one smaller marker with chromosome 18 material (Figure 4B). The metacentric marker had 18p material on one of the arms, positive for both 18p YACs. The smaller marker was positive for the 18p YACs and had a break in the centromere, or very close to it. The idic(18) chromosome arms hybridized to the wcp18, whereas the central segment was positive for pcp18q. Further hybridizations with the 18p YACs verified the idic(18). The central segment was negative for YAC 766f9, the most proximal marker on the q arm. Thus, this triploid tumour had lost two copies of 18q11–qter and gained two copies of 18p.

### LPC11p and LPC11m

The cytogenetic analysis of both the primary tumour and its metastasis revealed three to four copies of add(18)(q12). The FISH analysis, however, disclosed a second derivative 18, in two copies, with a large insertion in 18p, and YAC hybridizations showed that the insertion point was between 694b3 and the telomere. Cytogenetically, this insertion was identified as an ins(18;X)(p11;q21q28). One more derivative, der(18), was positive for pcp18q and the two 18p YACs but negative for 766f9. The add(18)(q12) was positive for the 766f9 and 937h12 probes but negative for the 18q21–22 markers. In the LPC11p, the add(18)(q12), ins(18;X) and der(18) were present in four, two and one copy respectively. The two former abnormalities were found in three and two copies in LPC11m, whereas the der(18) was only seen occasionally in the metastasis. As both LPC11p and LPC11m were tetraploid, the former had lost two copies of 18q12–qter and gained two copies of 18pter–cen–q12 and one copy of 18pter–cen–q11, whereas the latter sample had lost two copies of 18q12–qter and gained one copy of 18pter–cen–q12.

### LPC12m

The cytogenetic analysis showed one dic(18;21)(q11;p12) and one normal chromosome 18. The FISH analysis also disclosed two markers with small segments of 18q material, identified only with the pcp18q. The dic(18;21) was negative for the 766f9 probe but positive for pcp18q, indicating a break in 18q11. On balance, this triploid tumour had lost one chromosome 18 and one copy of 18q11–qter.

© Cancer Research Campaign 1998

In summary, the combined cytogenetic and FISH results revealed loss of 18q material, consistently including 18q12–qter, in all tumours. Out of 13 identified breaks resulting in deletion of 18q, 11 occurred in 18q11. Over-representation of 18p was detected in six samples, always including the entire arm.

### DISCUSSION

The present results showing gain of 18p in almost half of the pancreatic carcinoma samples analysed (6 out of 13) agree well with the previous CGH studies of Solinas-Toldo et al (1996), Fukushige et al (1997) and Mahlamäki et al (1997), revealing 18p over-representation in 4 of 23, 8 of 18 and 5 of 18 informative cases respectively. Neither in the present series nor in the previous CGH studies were partial duplications of 18p seen, i.e. no specific 18p region can, as yet, be delineated as the target for the detected over-representation. Gains of 18p have also been described in transitional cell carcinoma of the bladder, with a distinct amplification of 18p11 (Voorter et al, 1995). The oncogene *YES*, a SRC-related membrane-bound tyrosine kinase, is located within this band (Silverman et al, 1993) and has been found to be overexpressed in both melanoma (Loganzo et al, 1993) and colon carcinoma (Park et al, 1993). It is possible that over-representation of 18p may increase the expression of genes on this arm, such as *YES*, of possible pathogenic importance in pancreatic carcinogenesis. In four of the present cases (LPC2p, LPC6p, LPC7m and LPC12m), 18p material was lost. In three of these, however, the 18p deletions occurred through loss of one entire chromosome 18, and it is likely that the concomitant 18q deletion was the pathogenetically important change in these cases.

All 13 samples investigated showed under-representation of 18q material – as loss of chromosome 18 in three cases, complete loss of 18q in three cases and partial deletions of 18q in seven cases. None of the tumours displayed a total absence of a tested YAC marker, i.e. nullisomy for 18q material was not seen. The observed frequent loss of 18q is in agreement with earlier cytogenetic (Johansson et al, 1992; Bardi et al, 1993; Griffin et al, 1994, 1995), CGH (Fukushige et al, 1997; Mahlamäki et al, 1997) and LOH studies (Hahn et al, 1995). In the ten cases in which losses of 18q occurred through breaks in the q arm, the breakpoints were localized using both YAC and pcp18q probes. Of the recorded 13 breaks in 18q, 11 were mapped to 18q11. The fact that the breakpoints clustered to at least two different subregions within this band, 18q11.1 and 18q11.2 (Figure 3), argues against the presence of a preferentially break-prone region induced by a local change in the chromatin, as has been suggested for the frequent breaks in 3p14 in pancreatic carcinoma (Shridhar et al, 1996). Furthermore, the clustering of breaks close to the centromere indicates that loss of genes in bands 18q11 and 18q12 are important in the development of pancreatic tumours.

*SMAD4* and *DCC* are located in 18q21 and both these genes have been implicated in pancreatic tumorigenesis (Höhne et al, 1992; Simon et al, 1994; Hahn et al, 1996). However, no inactivating mutations in *DCC* have been reported in pancreatic cancer, and the importance of this gene in neoplasia has recently been questioned (Fazeli et al, 1997). *SMAD4*, on the other hand, has been shown to be functionally inactivated in a large proportion of pancreatic carcinomas (Hahn et al, 1996). However, *SMAD4* is only mutated in 22% of the cases showing LOH at this locus (Hahn et al, 1996), indicating that this gene may not always be the target for 18q deletions. The closely linked *SMAD2*, which shows a great sequence similarity to *SMAD4* and also functions as part of the transforming growth factor beta (TGFB) signal transducing pathway (Eppert et al, 1996), may, in analogy with *SMAD4*, function as a tumour-suppressor gene, although no *SMAD2* mutations in pancreatic carcinomas have been reported as yet. Taking into account the high frequency, 90–100%, of 18q deletions reported in pancreatic tumours (Hahn et al, 1995; Gorunova et al, 1998; this report), the relatively low incidence of functional abrogation of *SMAD4* in cases with LOH at this locus, and the present identification of proximal 18q deletion breakpoints, it seems reasonable to suggest the presence of an additional TSG on 18q of importance in pancreatic carcinomas. In favour of yet another TSG on 18q are recent molecular genetic findings in colon and lung carcinomas. Colon cancers show a similar deletion profile of 18q, with frequent breaks in 18q11–12, as pancreatic carcinoma, but with few concomitant inactivating mutations of *SMAD4* or *SMAD2* (Thiagalingam et al, 1996). Similarly, in lung cancer, which shows LOH at 18q21 in 30–60% (Schutte et al, 1996), functional loss of *SMAD4* or *SMAD2* is very rare (Nagatake et al, 1996; Uchida et al, 1996). Furthermore, mutations of *SMAD4* and *SMAD2* are rarely seen in other tumours, such as carcinomas of the oesophagus, stomach, bladder, prostate and breast showing LOH at 18q21 (Barrett et al, 1996; Lei et al, 1996; Schutte et al, 1996). We thus conclude that 18q harbours at least one more proximally located TSG, which is shared among various tumour types, including pancreatic tumours, with 18q deletions, and one, *SMAD4*, that may be specific for pancreatic cancer.

### ACKNOWLEDGEMENTS

This work was supported by the Swedish Cancer Society and the Gunnar, Arvid and Elisabeth Nilsson Foundation.

### REFERENCES

Bardi G, Johansson B, Pandis N, Mandahl N, Bak-Jensen E, Andrén-Sandberg Å, Mitelman F and Heim S (1993) Karyotypic abnormalities in tumours of the pancreas. *Br J Cancer* **67**: 1106–1112

Barrett MT, Schutte M, Kern SE and Reid BJ (1996) Allelic loss and mutational analysis of the *DPC4* gene in esophageal adenocarcinoma. *Cancer Res* **56**: 4351–4353

Bartsch D, Shevlin DW, Tung WS, Kisker O, Wells Jr SA and Goodfellow PJ (1995) Frequent mutations of *CDKN2* in primary pancreatic adenocarcinomas. *Genes Chrom Cancer* **14**: 189–195

Bray-Ward P, Menninger J, Lieman J, Desai T, Mokady N, Banks A and Ward DC (1996) Integration of cytogenetic, genetic, and physical maps of the human genome by FISH mapping of CEPH YAC clones. *Genomics* **32**: 1–14

Caldas C, Hahn SA, Da Costa LT, Redston MS, Schutte M, Seymour AB, Weinstein CL, Hruban RH, Yeo CJ and Kern SE (1994) Frequent somatic mutations and homozygous deletions of the p16 (*MTS1*) gene in pancreatic adenocarcinoma. *Nature Genet* **8**: 27–32

Eppert K, Scherer SW, Ozcelik H, Pirone R, Hoodless P, Kim H, Tsui L-C, Bapat B, Gallinger S, Andrulis IL, Thomsen GH, Wrana JL and Attisano L (1996) MADR2 maps to 18q21 and encodes a TGFβ-regulated MAD-related protein that is functionally mutated in colorectal carcinoma. *Cell* **86**: 543–552

Fazeli A, Dickinson SL, Hermiston ML, Tighe, RV, Steen RG, Small CG, Stoeckli E, Keino-Masu K, Masu M, Rayburn H, Simons J, Bronson RT, Gordon JI, Tessier-Lavigne, M and Weinberg RA (1997) Phenotype of mice lacking functional *Deleted in colorectal cancer (Dcc)* gene. *Nature* **386**: 796–804

Fearon, ER, Cho KR, Nigro JM, Kern SE, Simons JW, Ruppert JM, Hamilton SR, Preisinger AC, Thomas G, Kinzler KW and Vogelstein B (1990) Identification of a chromosome 18q gene that is altered in colorectal cancers. *Science* **247**: 49–56

Fukushige S, Waldman FM, Kimura M, Abe T, Furukawa T, Sunamura M, Kobari M and Horii A (1997) Frequent gain of copy number on the long arm of chromosome 20 in human pancreatic adenocarcinoma. *Genes Chrom Cancer* **19**: 161–169

© Cancer Research Campaign 1998

Giacalone J, Li X, Lehrach H and Francke U (1996) High-density radiation hybrid map of human chromosome 18 and contig of 18p. *Genomics* **37**: 9–18

Goggins M, Schutte M, Lu J, Moskaluk CA, Weinstein CL, Petersen GM, Yeo CJ, Jackson CE, Lynch HT, Hruban RH and Kern SE (1996) Germline *BRCA2* mutations in patients with apparently sporadic pancreatic carcinomas. *Cancer Res* **56**: 5360–5364

Gorunova L, Johansson B, Dawiskiba S, Andrén-Sandberg Å, Jin Y, Mandahl N, Heim S and Mitelman F (1995) Massive cytogenetic heterogeneity in a pancreatic carcinoma: fifty-four karyotypically unrelated clones. *Genes Chrom Cancer* **14**: 259–266

Gorunova L, Höglund M, Andrén-Sandberg Å, Dawiskiba S, Jin Y, Mitelman F and Johansson B (1998) Cytogenetic analysis of panreatic carcinomas: intratumor heterogeneity and nonrandom pattern of chromosome aberrations. *Genes Chromosomes Cancer* (in press)

Griffin CA, Hruban RH, Long PP, Morsberger LA, Douna-Issa, F and Yeo CJ (1994) Chromosome abnormalities in pancreatic adenocarcinoma. *Genes Chromosom Cancer* **9**: 93–100

Griffin CA, Hruban RH, Morsberger LA, Ellingham T, Long PP, Jaffee EM, Hauda KM, Bohlander SK and Yeo CJ (1995) Consistent chromosome abnormalities in adenocarcinoma of the pancreas. *Cancer Res* **55**: 2394–2399

Hahn SA, Seymour AB, Hoque ATMS, Schutte M, Da Costa LT, Redston MS, Caldas, C, Weinstein CL, Fischer A, Yeo CJ, Hruban RH and Kern SE (1995) Allelotype of pancreatic adenocarcinoma using xenograft enrichment. *Cancer Res* **55**: 4670–4675

Hahn SA, Schutte M, Hoque ATMS, Moskaluk CA, Da Costa LT, Rozenblum E, Weinstein CL, Fischer A, Yeo CJ, Hruban RH and Kern SE (1996) *DPC4*, a candidate tumor suppressor gene at human chromosome 18q21.1. *Science* **271**: 350–353

Höglund M, Gorunova L, Andrén-Sandberg Å, Dawiskiba S, Mitelman F and Johanson B (1998) Cytogenetic and fluorescence in situ hybridization analysis of chromosome 19 aberrations in pancreatic carcinomas: frequent loss of 19p13.3 and gain of 19q13.1–13.2. *Genes Chrom Cancer* **21**: 8–16

Höhne MW, Halatsch M-E, Kahl GF and Weinel RJ (1992) Frequent loss of expression of the potential tumor suppressor gene DCC in ductal pancreatic adenocarcinoma. *Cancer Res* **52**: 2612–2619

Johansson B, Bardi G, Heim S, Mandahl N, Mertens F, Bakjensen E, Andrén-Sandberg Å and Mitelman F (1992) Nonrandom chromosomal rearrangements in pancreatic carcinomas. *Cancer* **69**: 1674–1681

Lei J, Zou T-T, Shi Y-Q, Zhou X, Smolinski KN, Yin J, Souza RF, Appel R, Wang S, Cymes K, Chan O, Abraham JM, Harpaz N and Meltzer SJ (1996) Infrequent DPC4 gene mutation in esophageal cancer, gastric cancer and ulcerative colitis-associated neoplasms. *Oncogene* **13**: 2459–2462

Lengauer C, Green ED and Cremer T (1992) Fluorescence in situ hybridization of YAC clones after Alu-PCR amplification. *Genomics* **13**: 826–828

Loganzo F, Dosik JS, Zhao Y, Vidal MJ, Nanus DM, Sudol M and Albino AP (1993) Elevated expression of protein tyrosine kinase c-Yes, but not c-Src, in human malignant melanoma. *Oncogene* **8**: 2637–2644

Mahlamäki EH, Höglund M, Gorunova L, Karhu R, Dawiskiba S, Andrén-Sandberg Å, Kallioniemi O-P and Johansson B (1997) Comparative genomic hybridization reveals frequent gains of 20q, 8q, 11q, 12p, and 17q and losses of 18q, 9p, and 15q in pancreatic cancer. *Genes Chromo Cancer* **20**: 383–391

Mertens F, Johansson B, Höglund M and Mitelman F (1997) Chromosomal imbalance maps of malignant solid tumors: a cytogenetic survey of 3185 neoplasms. *Cancer Res* **37**: 2765–2780

Nagatake M, Takagi Y, Osada H, Uchida K, Mitsudomi T, Saji S, Shimokata K, Takahashi T and Takahashi T (1996) Somatic *in vivo* alterations of the *DPC4* gene at 18q21 in human lung cancers. *Cancer Res* **56**: 2718–2720

Park J, Meisler AI and Cartwright CA (1993) c-Yes tyrosine kinase activity in human colon carcinoma. *Oncogene* **8**: 2627–2635

Ruggeri B, Zhang S-Y, Caamano J, Dirado M, Flynn SD and Klein-Szanto AJP (1992) Human pancreatic carcinomas and cell lines reveal frequent and multiple alterations in the p53 and Rb-1 tumor-suppressor genes. *Oncogene* **7**: 1503–1511

Scarpa A, Capelli P, Mukai K, Zamboni G, Oda T, Iacono C and Hirohashi S (1993) Pancreatic adenocarcinomas frequently show p53 gene mutations. *Am J Pathol* **142**: 1534–1543

Schutte M, Hruban RH, Hedrick L, Cho KR, Nadasdy GM, Weinstein CL, Bova GS, Isaacs WB, Cairns P, Nawroz H, Sidransky D, Casero Jr, RA, Meltzer PS, Hahn SA and Kern SE (1996) *DPC4* gene in various tumor types. *Cancer Res* **56**: 2527–2530

Seymour AB, Hruban RH, Redston M, Caldas C, Powell SM, Kinzler KW, Yeo CJ and Kern SE (1994) Allelotype of pancreatic adenocarcinoma. *Cancer Res* **54**: 2761–2764

Shridhar R, Shridhar V, Wang X, Paradee W, Dugan M, Sarkar, F, Wilke C, Glover TW, Vaitkevicius VK and Smith DI (1996) Frequent breakpoints in the 3p14.2 fragile site, *FRA3B*, in pancreatic tumors. *Cancer Res* **56**: 4347–4350

Silverman, GA, Kuo W-L, Taillon-Miller P and Gray JW (1993) Chromosomal reassignment: YACs containing both YES1 and thymidylate synthase map to the short arm of chromosome 18. *Genomics* **15**: 442–445

Simon B, Weinel R, Höhne M, Watz J, Schmidt J, Kortner G and Arnold R (1994) Frequent alterations of the tumor suppressor genes P53 and DCC in human pancreatic carcinoma. *Gastroenterology* **106**: 1645–1651

Solinas-Toldo S, Wallrapp C, Müller-Pillasch F, Bentz M, Gress T and Lichter P (1996) Mapping of chromosomal imbalances in pancreatic carcinoma by comparative genomic hybridization. *Cancer Res* **56**: 3803–3807

Thiagalingam S, Lengauer C, Leach FS, Schutte M, Hahn SA, Overhauser J, Willson JKV, Markowitz S, Hamilton SR, Kern SE, Kinzler KW and Vogelstein B (1996) Evaluation of candidate tumor suppressor genes on chromosome 18 in colorectal cancers. *Nature Genet* **13**: 343–346

Uchida K, Nagatake M, Osada H, Yatabe Y, Kondo M, Mitsudomi T, Masuda A, Takahashi T and Takahashi T (1996) Somatic in vivo alterations of the *JV18-1* gene at 18q21 in human lung cancers. *Cancer Res* **56**: 5583–5585

Voorter C, Joos S, Bringuier PP, Vallinga M, Poddighe P, Schalken J, Du Manoir S, Ramaekers F, Lichter P and Hopman A (1995) Detection of chromosomal imbalances in transitional cell carcinoma of the bladder by comparative genomic hybridization. *Am J Pathol* **146**: 1341–1354

© Cancer Research Campaign 1998